# Fifteen-minute consultation: an evidence-based approach to research without prior consent (deferred consent) in neonatal and paediatric critical care trials

**DOI:** 10.1136/archdischild-2015-309245

**Published:** 2015-10-13

**Authors:** Kerry Woolfall, Lucy Frith, Angus Dawson, Carrol Gamble, Mark D Lyttle, the CONNECT advisory group, Bridget Young

**Affiliations:** 1 Department of Psychological Sciences, University of Liverpool, Liverpool, UK; 2 Department of Health Service Research, University of Liverpool, Liverpool, UK; 3 Centre for Values, Ethics and the Law in Medicine (VELiM), School of Public Health, University of Sydney, Sydney, New South Wales, Australia; 4 Department of Biostatistics, University of Liverpool, Liverpool, UK; 5 Emergency Department, Bristol Royal Hospital for Children, Bristol, UK; 6 Faculty of Health and Applied Sciences, University of the West of England, Bristol, UK

**Keywords:** Accident & Emergency, Ethics, Evidence Based Medicine, Intensive Care, Patient perspective

## What do we mean by research without prior consent (deferred consent)?

Emergency research with critically unwell children is vital to make sure that the most ill and injured children benefit from evidence-based healthcare.^1^ Ethical guidance require that consent be sought from parents (or legal representatives) on behalf of their children^2^ before research is initiated, yet concerns about problems in seeking parents’ consent when their child is critically ill have been a significant barrier to conducting clinical trials.^3^ ^4^ Taking time out to seek informed consent before starting treatment will often be difficult to justify as delaying any intervention in an emergency could diminish a child's chances of recovery. Parents will usually be highly distressed in a critical care situation, and many will struggle to make an informed decision about research in the limited time available.

Many countries have legislated to permit variations to informed consent and allow progress in research to develop critical care treatments.^5–7^ While the details vary, a common feature is that informed consent is not requested before the patient receives the intervention being researched.^8^ In the USA, the Food and Drug Administration (FDA) Exception from Informed Consent (EFIC) essentially ‘waives’ informed consent, although practitioners must show that they have attempted to contact legal representatives and tried to provide the opportunity to ‘opt out’ of a trial.^5^ ^9^ The FDA's detailed guidance aims to assist researchers in implementing EFIC,^10^ ^11^ although the accompanying public consultation requirements have led to varied practice and costly delays in setting up trials.^12^

Across European Union countries, legislation^7^ ^13^ enables practitioners to conduct research without seeking prior informed consent from parents when certain conditions are met (see box 1 for UK example). No accompanying guidance has been made available to assist researchers in implementing the legislation. European legislation does not name this alternative to informed consent, but it is commonly called ‘deferred consent’. We would argue that this is a misnomer as a child will have already received an intervention as part of a trial before any information is given or consent is sought. Essentially permission is sought post-intervention to use data that have already been collected and consent for the child to continue to take part in the trial. These problems with the terminology have led to much discussion recently, leading to a move towards the term ‘research without prior consent’, as it more accurately reflects the process of consent seeking in critical care research. We will, therefore, use the latter term for the rest of this article. However, regardless of what terminology is used, research without prior informed consent can be seen as eroding the autonomy of parents and children and has been much debated.^14–18^

Box 1 **Research without prior consent (deferred consent) can be conducted when the following conditions are met**

1. Treatment is required urgently
2. Urgent action is required for the purposes of the trial
3. It is not reasonably practicable to obtain consent prospectively
4. An ethics committee has given approval to the procedure under which the action is taken.^13^

## Have any trials been conducted without prior consent?

Although a number of adult critical care trials have been conducted without prior consent over the last decade,^19^ ^20^ the CATheter infections in CHildren trial (CATCH)^21^ was one of the first UK paediatric trials to use this approach since legislation changes in 2008. Another National Institute for Health Research-funded multicentre trial investigating the Emergency treatment with Levetiracetam or Phenytoin in Status Epilepticus,^22^ ^23^ which is using research without prior consent, has recently opened to recruitment. The number of trials using this approach is expected to increase over the next few years. It will be important to share experiences of conducting these challenging trials to inform peer and Research Ethics Committee reviews.

## What do parents and practitioners think about research without prior consent?

In the CONsent methods in childreN's emergEncy medicine and urgent Care Trials (CONNECT) study, we found that many parents recruited to CATCH were momentarily shocked or surprised to discover that their child had been entered into a trial without their consent, although they did not voice this to practitioners.^24^ After hearing practitioners explain why research without prior consent is being used in their situation—that it enables vital research to take place in time-critical situations—parents’ initial concerns were dispelled. Practitioners’ explanations were important to parents and helped to reassure them that there were good reasons for doing research without prior consent. Gauging the right moment to approach parents to discuss research is important—we found that mistiming the approach could add to parents’ distress.

Despite its ‘do then ask’ sequence, parents with experience of research without prior consent told us they felt *their* decisions about their child's participation had been voluntary. CONNECT and other research^25^ ^26^ have shown that parents support research without prior consent and appreciate the reasons for using it as long as their child's safety is not compromised. However, parents’ support for this approach may have its limits and is related to what is being trialled. Most parents in CONNECT remarked that they would be concerned about not seeking prior consent in trials involving either ‘new’ drug interventions that were not already used in clinical care or other potentially significant changes in clinical practice.^23^ ^24^

Practitioners’ views on research without prior consent differed depending upon whether or not they had experience of this method.^27^ Practitioners with no experience of research without prior consent were concerned that it would be detrimental to the parent–practitioner relationship. In contrast, practitioners with experience of this approach described how families were receptive to the method as long as discussions were appropriately timed and conducted sensitively.

We drew on the CONNECT study findings in light of bioethical principles, including voluntariness, autonomy, non-maleficence and justice,^18^ ^28^ to produce guidance on approaches to critical care research without prior informed consent.

## When should I approach parents to discuss research without prior consent?

CONNECT guidance recommends explaining what has happened at the earliest *appropriate* opportunity, which is likely to be after the initial emergency situation has passed. In such clinical situations, parents often rapidly form a close relationship with the child's nurse. Consulting with nursing staff about the child's condition and how parents are coping will help gauge when is an appropriate time.

## How should I explain to parents that their child has been entered into research without their prior informed consent?

Ask a member of staff known to the family to introduce you. Start by asking parents how their child is doing and check whether it is a convenient time to discuss research. Discuss key aspects of the trial, showing parents (and children if appropriate—see below) where information can be found on the participant information leaflet, paying particular attention to the key points shown in box 2. Allow parents time to consider the information (overnight if possible) and to ask questions about the trial. While it may be important to seek permission to use data already collected and consent for continued participation in the trial (and any follow-up procedures) before the child is discharged from hospital, it is also important to allow time to explore parents’ views and understanding of the trial and follow-up procedures.

Box 2 **Key points to cover when discussing research without prior consent with parents**

- Why the research is being conducted and why their child's condition made him/her eligible for the trial.
- That it was not possible to seek consent before the research intervention was given because their child needed immediate treatment, and it was not safe to delay this.
- That their permission is being sought to use information and/or samples that have already been collected and for their child to continue in the research.
- Details of how the intervention is already used in clinical practice (if applicable), any changes to clinical practice and potential risks of being in the research.
- That the research has been approved by an independent research ethics committee whose role is to review research to protect the rights, safety and well-being of participants.
- How the research findings will inform future treatments for critically ill children.
- That parents are free to choose whether or not their child's information is used in the research and that their decision will not affect their child's care.
- Details of any follow-up procedures arising from the research (if applicable).
- Where further information can be found—for example, leaflets, website, principal investigator.

Be prepared to address concerns that participation may have contributed to a poor recovery. It may help to explain any potential risks associated with participation in the trial, that the intervention is already used in clinical practice (if applicable), and to indicate that the research has been approved by a Research Ethics Committee. It may also help to explain that nobody will know which treatment is the most effective until the trial has been completed (which may take a few years) and to offer parents the opportunity to speak to the principal investigator or senior member of the research team to discuss any concerns.

## Should I involve children in the discussion?

Although children (under 16 years) cannot legally provide consent for their own participation in a trial, decisions about research should be shared by children and their parents^29^ if their maturity, condition and cognitive capacity allows. Young people (aged 16–18 years)^30^ can legally provide their own consent for a trial, although this is often impossible in an emergency situation. When assent (for children) or consent (for young people) cannot be sought due to their clinical condition, provide a developmentally appropriate information sheet to help parents discuss the research with their child when they have recovered. Provide contact details so that parents or children can discuss any aspect of the trial with the research team at a later date if they wish.

## What should happen if a child dies before parents are approached about the trial?

Legislation^7^ ^13^ does not stipulate what should happen in circumstances where a child dies. As there is wide variability and complexity of parental feelings about research when a child has been enrolled in a study and subsequently died, a one-size-fits-all approach to discussing a clinical trial is unlikely to be sensitive to the needs of grieving parents.^31^ Although there are some exceptions,^25^ many bereaved parents wish to be informed about their child's involvement and provided with the opportunity to discuss having their child's data analysed in a trial.^23^ ^31^ Talking with recently bereaved parents about research in which their child was involved will almost certainly be very difficult for you and for parents. However, in the interests of openness and honesty it is important to offer the opportunity to do so, otherwise parents will have no knowledge of their child's participation in research, nor can their child's data be included in the analyses. As well as potentially biasing the findings,^32^ this could be contrary to what parents would want for their child's data. Box 3 outlines some options to help those involved decide how to approach bereaved parents to discuss research without prior consent.

Box 3 **Options to consider when a child has died**

Option 1: Approach parents to explain about the trial before they leave hospital

- Discuss the trial and provide information before parents leave hospital. However, only approach parents with information and seek permission to use data already collected at this point if it is believed that parents have the capacity to absorb information and make an informed decision.

Option 2: Explain about the trial by letter at a later date

- If it is not thought appropriate to explain about the trial or seek permission to use data already collected before parents leave the hospital, consult with clinical colleagues and bereavement counsellors to identify an appropriate time to contact parents via a posted letter. Sending the letter could be timed to coincide with the bereavement follow-up invitation.
- The covering letter, information leaflet and consent form should be designed and worded specifically for bereaved parents. It should be prepared at the trial design stage and written in close consultation with bereaved parents, bereavement specialists and relevant special interest groups (see recommendation 1).
- The covering letter should be personalised and, if possible, signed by a clinician known to the family. The letter should explain that, understandably, parents will often have questions about the research in the days, weeks or months after the loss of a child and invite them to contact the trial team to arrange for a telephone or face-to-face discussion with the principal investigator about the trial if they wish. Include the bereaved parent information leaflet, consent form and stamped addressed envelope.
- At the outset of the trial, ethical approval may have been sought to include the anonymised data of deceased patients in analyses should no consent form be received from bereaved parents. Therefore, letters to parents should explain whether or not their child's data will be included in the trial if parents do not respond to the letter.
- Copies of the letters and accompanying documents sent to parents should be placed in the patient's notes.
- Be prepared to respond to parents who are concerned that research participation may have contributed to their child's death. Be careful to avoid giving false reassurance that this is not the case, unless it has been established by the principal/chief investigator that the cause of death was not related to the trial.

Option 3: Contact parents by telephone or letter to arrange a face-to-face discussion about the trial

- If it is not thought appropriate to explain about the trial or seek permission to use data already collected before parents leave the hospital, consult with clinical colleagues and bereavement counsellors to identify an appropriate time to contact parents via telephone or letter to arrange a face-to-face visit to discuss research.
- The letter should be personalised, signed by a clinician (known to the family if possible) and include a bereaved parent information leaflet.
- Copies of letters sent to parents should be placed in the patients’ notes.
- Provide parents with options for meeting location (eg, at their home or local hospital) as some parents may not wish to return to the hospital where their child died.
- During face-to-face discussions, explore parents’ views and understanding of the trial and why consent was not sought so that any concerns can be addressed.
- Be prepared to respond to parents who are concerned that trial participation may have contributed to their child's death. Be careful to avoid giving false reassurance that the trial did not contribute to their child's death unless it has been established by the principal/chief investigator that the cause of death was not related to the trial.
- If parents do not wish to have a face-to-face meeting, inform them that a trial information leaflet and consent form will be sent via post (see option 2).

## Conclusions

The CONNECT guidance will help practitioners to conduct research without prior consent in a way that is ethically appropriate and addresses the needs of families. Full CONNECT guidance can be found at https://www.liv.ac.uk/psychology-health-and-society/research/connect/ and will be reviewed and updated as further evidence becomes available. Research is required to look at the transferability of CONNECT guidance to other study types and settings, including adult critical care.
